# Bioaccumulation of Trace Elements in *Ruditapes philippinarum* from China: Public Health Risk Assessment Implications

**DOI:** 10.3390/ijerph10041392

**Published:** 2013-04-02

**Authors:** Feng Yang, Liqiang Zhao, Xiwu Yan, Yuan Wang

**Affiliations:** College of Fisheries and Life Science, Dalian Ocean University, Dalian 116023, China; E-Mails: yangfeng@dlou.edu.cn (F.Y.); zhaoliqiang@dlou.edu.cn (L.Z.); wangyuan@dlou.edu.cn (W.Y.)

**Keywords:** *Ruditapes philippinarum*, trace element, public health, risk assessment, China

## Abstract

The Manila clam *Ruditapes philippinarum* is one of the most important commercial bivalve species consumed in China. Evaluated metal burden in bivalve molluscs can pose potential risks to public health as a result of their frequent consumption. In this study, concentrations of 10 trace elements (Cu, Zn, Mn, Se, Ni, Cd, Cr, Pb, Hg and As) were determined in samples of the bivalve *Ruditapes philippinarum*, collected from nine mariculture zones along the coast of China between November and December in 2010, in order to evaluate the status of elemental metal pollution in these areas. Also, a public health risk assessment was untaken to assess the potential risks associated with the consumption of clams. The ranges of concentrations found for Cu, Zn, Mn, Se, Ni, Cd, Cr, Pb, Hg and As in *R. philippinarum* were 12.1–38.0, 49.5–168.3, 42.0–68.0, 4.19–8.71, 4.76–14.32, 0.41–1.11, 0.94–4.74, 0.32–2.59, 0.03–0.23 and 0.46–11.95 mg·kg^−1^ dry weight, respectively. Clear spatial variations were found for Cu, Zn, Cr, Pb, Hg and As, whereas Mn, Se, Ni, and Cd did not show significant spatial variation. Hotspots of trace element contamination in *R. philippinarum* can be found along the coast of China, from the north to the south, especially in the Bohai and Yellow Seas. Based on a 58.1 kg individual consuming 29 g of bivalve molluscs *per* day, the values of the estimated daily intake (EDI) of trace elements analyzed were significantly lower than the values of the accepted daily intake (ADI) established by Joint Food and Agriculture Organization/World Health Organization Expert Committee on Food Additives (JFAO/WHO) and the guidelines of the reference does (RfD) established by the United States Environmental Protection Agency (USEPA). Additionally, the risk of trace elements to humans through *R. philippinarum* consumption was also assessed. The calculated hazard quotients (HQ) of all trace elements were less than 1. Consequently, there was no obvious public risk from the intake of these trace elements through *R. philippinarum* consumption.

## 1. Introduction

The rapid development of industry and agriculture has been coupled with increasing environmental pollution, especially in developing countries [1]. As a consequence of these enhanced anthropogenic activities, a large amount of harmful pollutants such as persistent organic pollutants and heavy metals have been discharged into the estuarine and coastal waters over the past decades, which have placed increasing pressures on the corresponding estuarine and coastal ecosystems [2]. The input of these toxic wastes into the estuarine and coastal waters can result in deleterious effects on wildlife habitats, degradation of aquatic ecosystems and potential human health risks [3,4,5,6].

Among these harmful pollutants, much attention has been focused on heavy metal pollution in the estuarine and coastal waters [7,8,9]. Heavy metals, generally classified as non-biodegradable pollutants, can be strongly accumulated in surface sediments and aquatic organisms, and subsequently be transferred to humans through the food chain [10,11]. As is well-known, heavy metals, even at trace levels, can lead to adverse human health effects, including neurological diseases and cancers [12,13]. Consequently, researchers have classified heavy metals in the highest risk category for human health. A recent review reported that industrial and domestic sewage discharges, mining, smelting and *e*-wastes recycling are the main sources contributing to metal pollution in estuarine and coastal waters in China [6]. In recent years, very high metal concentrations in seawater, surface sediments and aquatic organisms collected from heavily industrialized areas have been intensively reported in China [14,15,16]. Consequently, the public health problems associated with consumption of contaminated seafood are of great interest today. Elevated heavy metal contamination in estuarine and coastal waters has increased the risk of heavy metal exposure to humans through consumption of contaminated seafood.

The Manila clam *Ruditapes philippinarum* is one of the most important commercial bivalve species consumed in China, and is an important seafood for humans due to its high nutritional value and delicate flavor. It is widely distributed in estuarine and coastal waters and has been cultivated for more than 50 years in China. Clam farming, particularly along the northern coast of China, has become an important component of the marine fishery industry [17]. The production of this species has reached levels of over 3.0 million tons per annum in China, which accounts for approximately 70% of mudflat fishery production and 90% of total World production [18]. Furthermore, they are also widely used as sentinel species for monitoring the quality of estuarine and coastal waters. As filter-feeding bivalves, they are well known to accumulate heavy metals from seawater, sediments and ingested food materials to concentrations greatly in excess of that found in their adjacent environments [19,20,21]. Additionally, they also conform to some desirable criteria such as widespread distribution, a sedentary lifestyle and ease of collection, making them the prime candidate for studying the heavy metal contamination in estuarine and coastal waters [22].

This study represents a preliminary investigation of levels of ten trace elements (Cu, Zn, Mn, Se, Ni, Cd, Cr, Pb, Hg and As) in Manila clam samples collected along the coast of China. The primary purpose of our study was to examine whether this bivalve species was contaminated with these selected trace elements. Potential human health risk assessment was then conducted to evaluate whether it presented any potential hazard to humans as a result of consumption. From a public health point of view, our study will provide consumers with better knowledge of public health in connection with seafood consumption.

## 2. Methods

### 2.1. Sampling

**Figure 1:**
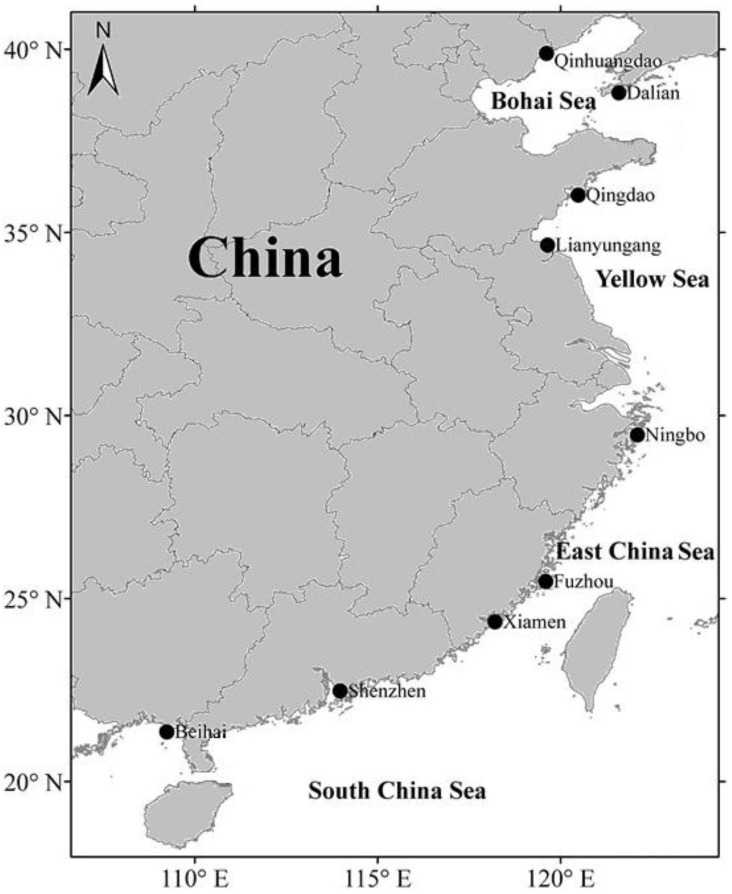
Map of the sampling sites along the coast of China.

Manila clams were collected from nine sampling sites along the coast of China between November and December in 2010 (Figure 1).

These collected samples were transferred to the Biological Laboratory of Dalian Ocean University and cultured in filtered seawater for 24 h depuration. 10 individuals from each site, with similar size, weight and shape, were pooled to prepare a composite specimen in order to minimize individual variations in trace elements determined. Differences in the shell length and wet weight of samples among all sampling sites were not statically significant. Three replicates were performed at each sampling site. The whole soft tissues of clams were dissected with a plastic knife, rinsed five times with deionized water (resistivity, 18.2 MΩ/cm; Millipore, Co., Bedford, MA, USA), freeze-dried, homogenized and then stored in polyethylene bags at −20 °C until future analysis.

### 2.2. Trace Element Analysis

Approximated 0.5 g of dry sample was digested with 6 mL of HNO_3_ (65%) and 2 mL of H_2_O_2_ (30%) in a microwave digestion system (MARSX, CEM, Mathews, NC, USA) for 30 min. After cooling, the resultant solution was diluted to 50 mL with deionized water. Concentrations of Cu, Zn, Mn, Se, Ni, Cd, Cr and Pb were determined by inductively coupled plasma atomic emission spectrometer (ICP-AES, IRIS intrepid 11XSP, Thermo Electron Co., San Jose, CA, USA), while Hg and As were determined by atomic fluorescence spectrometer (AFS9130, Beijing Titan Instruments Co. Ltd., Beijing, China). Standard reference materials (mussel tissue sample, GBW08571, from the National Research Center for Certified Reference Materials, Beijing, China) were used for quality assurance and quality control procedures. The results showed good agreement between the certified and the analytical values. All trace elements concentrations were given on a dry weight basis (mg·kg^−^^1^ dry weight).

### 2.3. Human Exposure Assessment

The potential human health risk assessment was conducted by considering the following parameters according to Onsanit *et al.* [23]. The estimated daily intake (EDI), provisional tolerance weekly intake (PTWI), accepted daily intake (ADI), and reference dose (RfD) were previously established by the Joint FAO/WHO Expert Committee on Food Additives (JECFA) [24], and the United States Environmental Protection Agency [25]. The EDI (mg/kg bw/day) was calculated using the following equation:

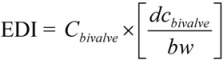
(1)
where *C_bivalve_* = average trace metal concentrations in *R. philippinarum* (mg·kg^−^^1^ wet weight), *dc_bivalve_* = daily bivalve consumption (g·day^−^^1^) *per capita* for the Chinese population as recorded by the FAO [26], and *b_W_* = average body weight (kg) of the target population [27].

The hazard quotient (HQ) was calculated by dividing the EDI by the established RfD to assess human risk associated with bivalve consumption. There would be no obvious hazard if the value of HQ was less than 1. The HQ was calculated using the following equation:

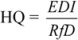
(2)

## 3. Results

### 3.1. Geographical Distribution of Trace Elements in Clams

**Figure 2:**
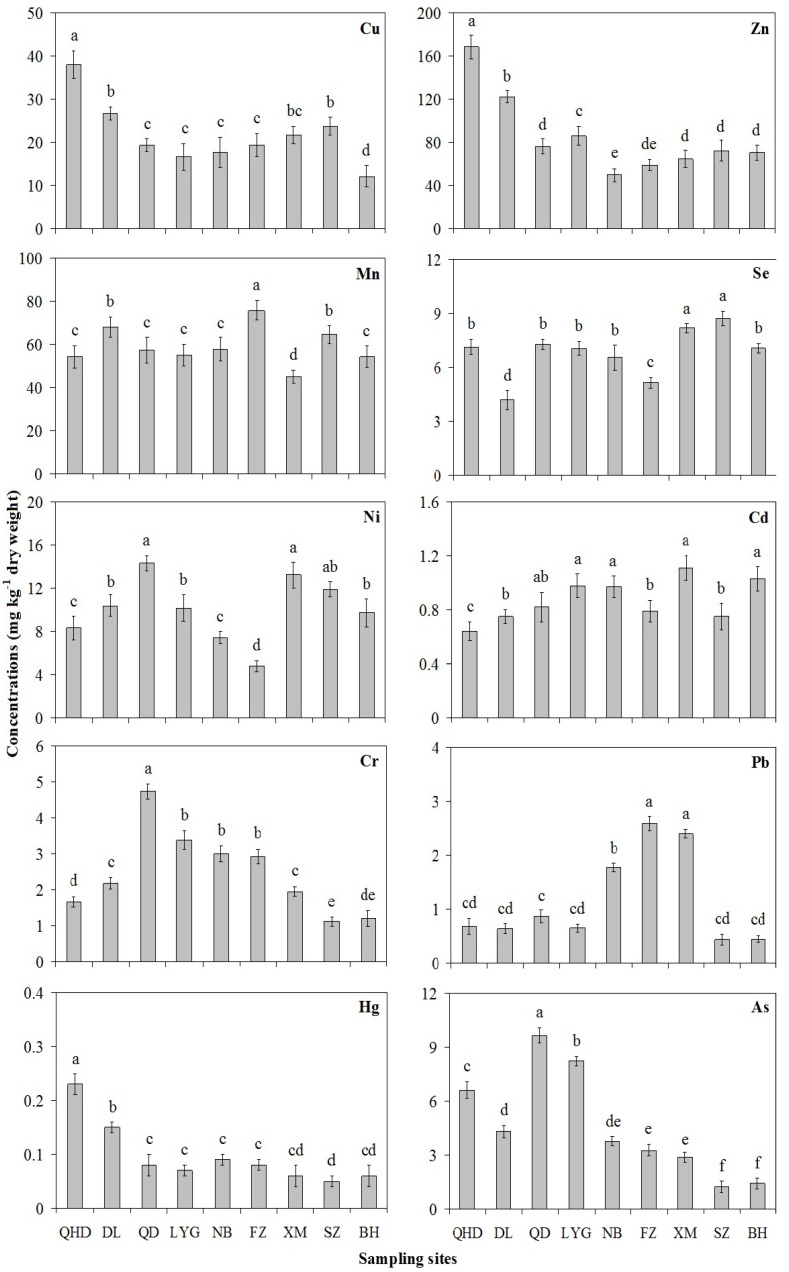
Trace element concentrations (mg·kg^−1^ dry weight) in *Ruditapes philippinarum* along the coast of China. Values with different marks represent significant difference at the significance level of 0.05. Data are expressed as mean ±SD (n = 3). QHD: Qinghuangdao; DL: Dalian; QD: Qingdao; LYG: Lianyungang; NB: Ningbo; FZ: Fuzhou; XM: Xiamen; SZ: Shenzheng; BH: Beihai.

Trace element concentrations in clams collected from sampling sites along the coast of China are shown in Figure 2. In general, concentrations of selected trace elements in clams were consistent and ranked, in decreasing order of concentration, as follows: Zn > Mn > Cu > Ni > Se > As > Cr > Cd > Pb > Hg. Differences in trace element concentrations from different sampling sites are described below.

Zn, Mn, Cu, and Se are well-known essential trace elements for all living organisms. As shown in Figure 2, Zn was the most prominent element detected in *R. philippinarum*, with the lowest level, of 49.5 mg·kg^−^^1^, being found in Ningbo and the highest level, of 168.3 mg·kg^−^^1^, occurring in Qinghuangdao. The minimum and maximum Mn levels were found to be 45.0 mg·kg^−^^1^ in Xiamen, and 68.0 mg·kg^−^^1^ in Dalian, respectively, while the lowest and highest Cu levels were 12.1 mg·kg^−^^1^ in Beihai, and 38.0 mg·kg^−^^1^ in Qinghuangdao, respectively. The lowest and highest levels of Se were, respectively, 4.19 mg·kg^−^^1^ in Dalian, and 8.71 mg·kg^−^^1^ in Shenzheng. Ni and Cr have been identified as potential essential metals. The lowest and highest levels of Ni were 4.76 mg·kg^−^^1^ in Fuzhou, and 14.32 mg·kg^−^^1^ in Qingdao, respectively, while the minimum and maximum Cr levels were found to be 1.12 mg·kg^−^^1^ in Shenzheng, and 4.74 mg·kg^−^^1^ in Qingdao, respectively.

As, Cd, Pb and Hg have been classified as potentially toxic elements due to their persistence, high bioavailability, and high toxicity. The lowest and highest As levels in clams were, respectively, 0.62 mg·kg^−^^1^ in Shenzheng, and 9.66 mg·kg^−^^1^ in Qingdao. The lowest and highest Cd levels were 0.64 mg·kg^−^^1^ in Qinghuangdao and 1.11 mg·kg^−^^1^ in Xiamen, respectively. The minimum and maximum Pb levels were found to be 0.44 mg·kg^−^^1^ in Shenzheng, and 2.59 mg·kg^−^^1^ in Fuzhou, respectively, while the lowest and highest Hg levels were, respectively, 0.05 mg·kg^−^^1^ in Shenzheng, and 0.23 mg·kg^−^^1^ in Qinghuangdao.

### 3.2. Estimated Exposure and Hazards Quotients of Trace Elements

The mean trace element concentrations were used to estimate the daily intake of trace elements through *R. philippinarum* consumption by the Chinese population. JFAO/WHO have set a PTWI of Cu as 3.5 mg·kg^−1^ bw, a PTWI of Zn as 1.5 mg·kg^−1^ bw, a PTWI of Mn as 0.98 mg·kg^−1^ bw, a PTWI of Se as 0.035 mg·kg^−1^ bw, a PTWI of Cd as 0.007 mg·kg^−1^ bw, a PTWI of Pb as 0.025 mg·kg^−1^ bw, a PTWI of Hg as 0.005 mg·kg^−1^ bw and a PTWI of As as 0.015 mg·kg^−1^ bw, respectively. Thus the values of ADI (mg·kg^−1^ bw·day^−1^) calculated from PTWI were 0.5 for Cu, 0.21 for Zn, 0.14 for Mn, 0.005 for Se, 0.001 for Cd, 0.0036 for Pb, 0.0007 for Hg and 0.0021 for As, respectively. An average weight of 58.1 kg was assumed for a Chinese person based on statistics from 158,666 Chinese people from all provinces [27]. On average, the daily consumption rate of bivalves is 29 g/person/day in China [26]. The toxic effects of As depend on the chemical forms present, and it is only toxic if present in inorganic forms, such as arsenate and arsenite [28]. About 10% of total As in marine organisms is present in inorganic forms [29]. Thus only inorganic As was considered in the hazard calculation. On this basis, the values of estimated daily intake (EDI) and hazard quotient (HQ) of trace elements are shown in Table 1. The calculated HQs of Cu, Zn, Mn, Se, Ni, Cd, Cr, Hg, and As in our study (except for Pb, which did not have an RfD value) were all less than 1.

**Table 1:** Estimateddaily intake (EDI) (mg·kg^−1^ bw·day^−1^)and hazard quotients (HQs) of trace elements through *Ruditapes philippinarum* consumption by people in China. Data reported based on wet weight are converted to dry weight basis for comparison using a wet weight/dry weight ratio of 7. PTWI (mg·kg^−1^ bw): provisional tolerance weekly; ADI (mg·kg^−1^ bw·day^−1^): Acceptable daily intake; Average concentrations of inorganic As were estimated as 10% of total As. n.a.: no available data.

Location		Cu	Zn	Mn	Se	Ni	Cd	Cr	Pb	Hg	As
Qinghuangdao	EDI	0.002710	0.012001	0.003872	0.000509	0.000592	0.000046	0.000118	0.000048	0.000016	0.000047
	HQ	0.0678	0.0400	0.0277	0.1018	0.0118	0.0460	0.0393	n.a.	0.0533	0.1175
Dalian	EDI	0.001904	0.008721	0.004849	0.000299	0.000739	0.000053	0.000155	0.000046	0.000011	0.000031
	HQ	0.0476	0.0291	0.0346	0.0598	0.0148	0.0530	0.0517	n.a.	0.0367	0.0775
Qingdao	EDI	0.001376	0.005441	0.004086	0.000519	0.001021	0.000058	0.000338	0.000061	0.000006	0.000069
	HQ	0.0344	0.0181	0.0292	0.1038	0.0204	0.0580	0.1127	n.a.	0.0200	0.1725
Lianyungang	EDI	0.001184	0.006132	0.003922	0.000503	0.000724	0.000070	0.000241	0.000046	0.000005	0.000059
	HQ	0.0296	0.0204	0.0280	0.1006	0.0145	0.0700	0.0803	n.a.	0.0167	0.1475
Ningbo	EDI	0.001262	0.003530	0.004114	0.000466	0.000530	0.000069	0.000214	0.000127	0.000006	0.000027
	HQ	0.0316	0.0118	0.0294	0.0932	0.0106	0.0690	0.0713	n.a.	0.0200	0.0675
Fuzhou	EDI	0.001376	0.004207	0.005398	0.000367	0.000339	0.000056	0.000208	0.000185	0.000006	0.000023
	HQ	0.0344	0.0140	0.0386	0.0734	0.0068	0.0560	0.0693	n.a.	0.0200	0.0575
Xiamen	EDI	0.001547	0.004613	0.003209	0.000583	0.000944	0.000079	0.000139	0.000171	0.000004	0.000021
	HQ	0.0387	0.0154	0.0229	0.1166	0.0189	0.0790	0.0463	n.a.	0.0133	0.0525
Shenzheng	EDI	0.001690	0.005155	0.004613	0.000621	0.000792	0.000053	0.000080	0.000031	0.000004	0.000004
	HQ	0.0423	0.0172	0.0330	0.1242	0.0158	0.0530	0.0267	n.a.	0.0133	0.0100
Beihai	EDI	0.000863	0.005041	0.003872	0.000504	0.000695	0.000073	0.000086	0.000032	0.000004	0.000005
	HQ	0.0216	0.0168	0.0277	0.1008	0.0139	0.0730	0.0287	n.a.	0.0133	0.0125
PTWI		3.5	1.5	0.98	0.035	n.a.	0.007	n.a.	0.025	0.005	0.015
ADI		0.5	0.21	0.14	0.005	n.a.	0.001	n.a.	0.0036	0.0007	0.0021
RfD		0.04	0.3	0.14	0.005	0.05	0.001	0.003	n.a.	0.0003	0.0004

## 4. Discussion and Conclusions

### 4.1. Geographical Variance

Environmental factors such as metal concentration, speciation, food quality and quantity can be critical in influencing the metal accumulation in bivalves living in different environments [6]. In general, metals were heterogeneously distributed in bivalves and formed hot spot along the coastal areas. In our study, we discuss the geographical variance of trace elements in *R. philippinarum*, from the north to the south, by splitting the Chinese coastal areas into four subsections—Bohai Sea, Yellow Sea, East China Sea, and South China Sea (Figure 1).

It is well known that the Bohai Sea includes the most contaminated coastal areas in China. The fishery resource in the Bohai Sea has declined dramatically in the past twenty years because of overexploitation, eutrophication, and contamination [6]. Alarmingly high trace element levels were observed in *R. philippinarum* from the Bohai Sea (Table 2). Wang reported that the highest concentrations of Cu, Zn, Mn, Se, Ni, Cd, Cr, Pb, Hg and As in *R. philippinarum* were 25.7, 190.5, 94.8, 23.10, 14.25, 3.71, 19.1, 2.00, 0.27 and 14.25 mg·kg^−^^1^, respectively [15]. Bioaccumulation of Cd, Co, Cu, Ni, Pb, and Zn had obviously occurred in *R. philippinarum* from the Bohai Sea [30]. In our study, concentrations of Cu, Zn, Mn, Se, Ni, Cd, Cr, Pb, Hg, and As in *R. philippinarum* from Qinghuangdao and Dalian ranged from 26.7–38.0, 122.3–168.3, 54.3–68.0, 4.19–7.14, 8.30–10.37, 0.64–0.75, 1.66–2.18, 0.64–0.68, 0.15–0.23 and 4.31–6.62 mg·kg^−^^1^, respectively. *R. philippinarum* may be considerably contaminated by Cu, Zn, and Hg in the Bohai Sea. Zheng reported that the most contaminated areas along the Bohai Sea coastline is where the Huludao Zinc Plant is located, which is the largest zinc plant in Asia [31]. Extremely high Cu and Zn concentrations were found in vegetables due to zinc smelting activities [32]. The primary area of Hg contamination from the Bohai Sea is in the northern region, which has been polluted by industrial wastewater and sewage discharge [33]. Trace element levels observed in bivalves consistently reflect the elevated metal concentrations in this environment. Therefore, we recommend a sustained bivalve-based monitoring program in the Bohai Sea to test for locational differences as a function of anthropogenic activities, and to prevent potential human health risks.

Jiaozhou Bay, one of the most important clam-producing areas in China, has been recognized as the prototypical case of metal contamination in the Yellow Sea. It is a semi-closed bay situated in the northern part of Shandong Peninsula in China. In the recent years, the environment of this area has undergone serious deterioration and this poses a threat to the clam production in the area [34]. Many studies have reported the current metal contamination in *R. philippinarum* collected from Jiaozhou Bay (Table 2). Wang reported that the maximum concentrations of Cu, Zn, Mn, Ni, Cd, Cr and Pb in *R. philippinarum* were 26.0, 110.3, 63.9, 52.75, 3.31, 35.47 and 14.77 mg·kg^−^^1^, respectively [18]. The highest Hg and As in *R. philippinarum* levels reached 0.21 and 15.54 mg·kg^−^^1^ [35]. Most of the highest trace element values occurred in bivalves collected from the northeast bay and the lowest values occurred in those collected from the western part [34,36]. In this study, concentrations of Cu, Zn, Mn, Se, Ni, Cd, Cr, Pb, Hg and As in *R. philippinarum* from Qingdao and Lianyungang ranged from 16.6–19.3, 76.3–86.0, 55.0–57.3, 7.05–7.28, 10.15–14.32, 0.82–0.98, 3.38–4.74, 0.65–0.86, 0.07–0.09 and 8.25–9.66 mg·kg^−^^1^, respectively. *R. philippinarum* from the Yellow Sea was significantly contaminated by Cr and As with the highest concentrations of 4.74 and 11.95 mg·kg^−^^1^, respectively. Similarly, concentrations of these trace elements were concordant with those reported from Jiaozhou Bay (Table 2).

The East China Sea is one of the world’s largest continental shelf systems, and receives the discharge from the Yangtze River that is the third largest river in the World [37]. Müller found that the metal levels in the suspended sediments from the Yangtze River were several times higher than those found in rivers in other countries, implying that significant quantities of metals are discharging into the East China Sea via this river [38]. Fung reported a mussel-based monitoring program, which was carried out along the East China Sea coast using *Perna viridis* and *Mytilus edulis*, and found that the concentrations of trace elements were, in general, higher or at least comparable to those reported in other regional studies [14]. On the contrary, Huang reported that Zhejiang coastal areas might be considered relatively unpolluted with trace elements and the concentrations of Hg, Cd, Pb, Zn, Cu, and As in bivalves also below the seafood safety limits for human consumption [16]. Concentrations of Cu, Zn, Mn, Se, Ni, Cd, Cr, Pb, Hg and As in *R. philippinarum* from Ningbo, Fuzhou, and Xiamen ranged from 17.7–21.7, 49.5–64.7, 45.0–75.7, 5.15–8.18, 4.76–13.24, 0.79–1.11, 1.95–3.00, 1.78–2.59, 0.06–0.09 and 2.90–3.79 mg·kg^−^^1^, respectively. Lead contamination in *R. philippinarum* appeared significant in the East China Sea, with levels obtained several times higher than in clams collected from other sites (Table 2). Lin reported that the mean concentration of dissolved Pb (~128 ng/L) in the southern East China Sea is approximately several times higher than those in the Pacific Ocean, and the high dissolved Pb levels in the southern East China Sea waters correspond to much higher atmospheric supplies of Pb to the East China Sea [39].

It is generally considered that marine pollution in the southern China is relatively lower than in the northern part. High levels of trace metals in the South China Sea are mainly because of the enhanced industrial activities along the south coast of China, especially in the Pearl River Estuary, which is known as one of the most industrialized and urbanized regions in China and acts as a major sink for contaminants and nutrients in the surrounding ecosystem [6]. Fang reported that the concentrations of trace elements in molluscs collected from the Pearl River Estuary were significantly higher than those collected from other sites along the South China Sea coast [40,41]. Concentrations of Cu, Zn, Mn, Se, Ni, Cd, Cr, Pb, Hg and As in *R. philippinarum* from Shenzheng and Beihai ranged from 12.1–23.7, 70.7–72.3, 54.3–64.7, 7.07–8.71, 9.74–11.11, 0.75–1.03, 1.12–1.20, 0.44–0.45, 0.05–0.06 and 0.62–0.72 mg·kg^−^^1^, respectively. As shown in Table 2, trace element concentrations in *R. philippinarum* from the South China Sea were lower than from those collected from the Bohai, Yellow and East China Seas.

**Table 2:** Comparison trace element concentrations (mg·kg^−1^ dry weight) in *Ruditapes philippinarum* obtained in the present study with literature data from China and other regions all over the world. n.a.: no available data.

Region	Cu	Zn	Mn	Se	Ni	Cd	Cr	Pb	Hg	As	Reference
Bohai Sea, China	20.0–38.0	122.3–168.3	47.5–68.0	4.19–7.14	8.30–12.26	0.64–0.75	1.66–2.29	0.64–0.72	0.15–0.23	4.31–6.62	This study
Yellow Sea, China	14.0–19.3	76.3–86.0	51.0–57.3	6.24–7.28	10.15–14.32	0.42–0.98	3.38–4.74,	0.65–0.86	0.07–0.09	8.25–11.95	This study
East China Sea, China	17.7–21.7	49.5–64.7	45.0–75.7	5.15–8.18	4.76–13.24	0.79–1.11	1.95–3.00	1.78–2.59	0.06–0.09	2.90–3.79	This study
South China Sea, China	12.1–23.7	50.7–72.3	42.0–64.7	6.21–8.71	8.40–9.74	0.41–1.03	0.94–1.25	0.32–0.54	0.03–0.06	0.46–0.85	This study
Bohai Sea, China	7.5–25.7	58.5–190.5	14.1–94.8	2.66–23.10	9.90–14.25	0.82–3.71	0.94–19.1	0.47–2.00	0.08–0.27	9.9–14.25	[15]
Bohai Sea, China	8.96–30.59	70.0–140.4	n.a.	n.a.	5.04–20.79	0.98–4.41	n.a.	0.91–2.38	n.a.	n.a.	[30]
Jiaozhou Bay, China	5.1–26.0	52.1–110.3	10.3–63.9	n.a.	5.26–52.75	0.65–3.31	9.64–35.47	0.89–14.77	n.a.	n.a.	[18]
Jiaozhou Bay, China	7.7–9.1	62.2–87.1	n.a.	n.a.	n.a.	0.39–0.68	0.96–4.04	0.53–1.42	0.04–0.07	8.32–14.33	[34]
Jiaozhou Bay, China	8.8–21.5	n.a.	n.a.	n.a.	n.a.	0.07–1.05	9.03–10.92	2.10–3.64	0.02–0.21	9.80–15.54	[35]
Jiaozhou Bay, China	6.4–19.8	35.5–85.5	27.45–67.6	n.a.	n.a.	0.51–0.67	n.a.	0.31–1.01	n.a.	n.a.	[36]
East China Sea, China	9.2–14.4	26.0–73.5	n.a.	n.a.	n.a.	0.48–0.85	n.a.	0.23–0.42	0.06–0.15	2.47–3.45	[16]
South China Sea, China	6.0–10.0	71.0–116.0	n.a.	n.a.	n.a.	0.60–5.00	0.20–14.50	0.80–2.70	n.a.	n.a.	[6]
Pearl River Delta, China	4.0–10.1	39.4–212.9	n.a.	n.a.	5.04–5.88	0.42–1.19	3.85–4.13	1.47–1.75	n.a.	n.a.	[40]
Pearl River Delta, China	5.2–13.9	54.9–74.6	n.a.	n.a.	9.78–23.72	0.30–7.60	3.13–12.25	1.50–14.99	n.a.	n.a.	[41]
Southern Atlantic, Spanish	5.1–26.0	52.1–110.3	10.3–63.9	n.a.	5.26–52.75	0.47–2.71	9.6–35.5	0.41–1.94	0.13–2.72	12.60–32.20	[9]
Gironde Estuary, France	9.4 ± 1.0	97.2 ± 8.0	n.a.	n.a.	n.a.	0.52 ± 0.11	n.a.	n.a.	n.a.	n.a.	[11]
Kyeonggi Bay, Korea	5.5–14.3	64.7–162.0	20.5–177.0	n.a.	2.74–33.50	0.53–2.20	0.61–2.38	0.34–1.72	n.a.	n.a.	[20]
Shihwa Lake, Korea	6.6–23.2	70.0–144.0	n.a.	n.a.	3.20–13.4	0.56–1.11	0.42–1.40	0.50–1.82	n.a.	12.4–18.3	[21]
Venice lagoon, Italy	8.2–29.0	60.0–122.0	8.0–29.1	n.a.	0.15–8.37	0.26–2.19	1.89–5.70	0.49–2.54	0.25–2.30	18.90–64.00	[22]

### 4.2. Public Health Risk Assessment

The estimated daily intake (EDI) of *R. philippinarum* lead to estimates of trace element consumption in the range of 1.3–68.3 times lower than the RfD guidelines for all trace elements studied, thus strongly indicating that people would not experience significant health risks from the intake of trace elements through clams *R. philippinarum* consumption along the coast of China. According to the FAO [26], the average seafood and fish consumption of Chinese people is 42 g/person/day, including freshwater fish 29 g/person/day, marine fish 3 g/person/day and shellfish 29 g/person/day. However, people living along the coast are expected to consume much more seafood than people living inland. In Hong Kong, for example, the estimated daily consumption of marine fish is 25 g/day (or eight times higher than the average Chinese consumption) [23]. In our study, daily clam consumption was assumed as the daily shellfish consumption of Chinese people. Even with such high clam consumption, the EDI was also lower than the established ADI. Our calculations suggested that the HQs of all trace elements examined in this study, except for Pb, which did not have the RfD value, were all less than 1, suggesting that the consumption of clams did not present any risk to humans. Nevertheless, attention should be given to As, as the relatively high HQ values of As in the present study indicated that the contamination of As in clams may present a potential human health risk.
